# Midlife diseases of despair and cardiometabolic risk: Testing shared origins in adolescent psychopathology – CORRIGENDUM

**DOI:** 10.1017/S0033291725101086

**Published:** 2025-07-16

**Authors:** Kallisse R. Dent, Grace M. Brennan, Lara Khalifeh, Leah S. Richmond-Rakerd

**Affiliations:** 1 Department of Psychology, University of Michigan, Ann Arbor, MI, USA; 2 Department of Psychology and Neuroscience, Duke University, Durham, NC, USA; 3 Duke Aging Center, Duke University School of Medicine, Durham, NC, USA

This article was published in Psychological Medicine with an error in the coding of the prospective assessment of depression via the Center for Epidemiological Studies Depression Scale (CES-D). Four items that should have been reverse-scored (“You felt that you were just as good as other people,” “You felt hopeful about the future,” “You were happy,” “You enjoyed life”) were not reverse-scored prior to constructing CES-D sum scores. Correcting this error results in a change in the weighted prevalence of adolescent psychopathology (previous estimate=30.3%, corrected estimate=25.5%; Results section 1, Table 1). In corrected analyses, there are no changes to our primary conclusions. Some of the associations previously identified are in fact strengthened with the corrected variable. The following is a summary, with reference to specific areas of the manuscript where estimates have changed:

1. Tests of associations of adolescent psychopathology with midlife diseases of despair and cardiometabolic risk (Abstract, Results sections 2 and 3, Figure 2, Table 2): In corrected analyses, we continue to identify statistically-significant associations of any adolescent psychopathology, internalizing psychopathology, and number of adolescent mental-health conditions with both outcomes, and effect sizes are increased. Corrected estimates of association for any adolescent psychopathology with diseases of despair and cardiometabolic risk: baseline incidence rate ratios (IRRs)=1.87 [1.66-2.09] and 1.20 [1.11-1.29], respectively; adjusted IRRs=1.81 [1.60-2.03] and 1.18 [1.09-1.27], respectively. Corrected estimates of association for internalizing psychopathology with diseases of despair and cardiometabolic risk: baseline IRRs=1.98 [1.74-2.22] and 1.22 [1.11-1.32], respectively; adjusted IRRs=1.91 [1.67-2.15] and 1.19 [1.09-1.30], respectively. Corrected estimates of association for number of adolescent mental-health conditions with diseases of despair and cardiometabolic risk: baseline IRRs=1.59 [1.48-1.71] and 1.15 [1.09-1.22], respectively; adjusted IRRs=1.56 [1.44-1.67] and 1.14 [1.08-1.20], respectively.
2. Tests of mediation of associations of adolescent psychopathology with midlife diseases of despair (Results section 5, Supplemental Table 7, Discussion paragraph 4): In corrected analyses, consistent with previous findings, low education is a statistically-significant mediator, but explains only a very small proportion of the association (previous estimate=2.1%, corrected estimate=2.0%). Consistent with previous findings, mediation by early-adult substance use is statistically significant, and the estimate of percent mediation is unchanged (21.5%). Statistically-significant mediation by early-adult substance use also still persists after excluding substance use from the midlife diseases of despair outcome (previous estimate=10.3%, corrected estimate=10.6%). Also consistent with previous findings, in corrected analyses we continue to observe minimal mediation of associations of adolescent psychopathology with midlife diseases of despair by early-adult physical inactivity, fast-food consumption, and healthcare management. Previously, early-adult healthcare management was not a statistically-significant mediator. In corrected analyses, the mediation effect is statistically significant, but it explains only 1.2% of the association.
3. Tests of mediation of associations of adolescent psychopathology with midlife cardiometabolic risk (Results section 5, Supplemental Table 8): In corrected analyses, consistent with previous findings, we find no evidence of statistically-significant mediation by any variables.
4. Tests of the extent to which adolescent psychopathology accounts for the co-occurrence of midlife diseases of despair and cardiometabolic risk (Results section 6, Table 3, Discussion paragraph 5): In corrected analyses, the percentage of the association between diseases of despair and cardiometabolic risk accounted for by adolescent psychopathology is increased, from 8.3% to 16.7% (corrected IRR adjusted for adolescent psychopathology=1.10 [1.06-1.14]). The percentage of the association accounted for by adolescent psychopathology together with adolescent SES and adolescent cognitive ability is increased from 16.7% to 25.0%. Consistent with previous findings, the association between midlife diseases of despair and cardiometabolic risk is statistically significant even after accounting for adolescent psychopathology, adolescent SES, and adolescent cognitive ability (corrected IRR adjusted for all three factors=1.09 [1.05-1.13]).
  In Discussion paragraph 5, we previously interpreted this finding as follows: “In middle adulthood, individuals with more diseases of despair also tended to experience more indicators of cardiometabolic risk. However, despite the associations of adolescent psychopathology with each outcome individually, adolescent psychopathology explained a somewhat modest degree (approximately eight percent) of their co-occurrence. As such, many adolescents with mental-health problems tended to develop *either* more indicators of despair-related diseases or cardiometabolic risk, but not both….” We suggest the following revised language to take into account the larger degree of co-occurrence accounted for by adolescent psychopathology in corrected analyses: “In middle adulthood, individuals with more diseases of despair also tended to experience more indicators of cardiometabolic risk. Adolescent psychopathology explained a moderate degree (approximately seventeen percent) of their co-occurrence. This suggests that some adolescents with mental-health problems went on to develop more indicators of both midlife diseases of despair and cardiometabolic risk. However, many adolescents with mental-health problems developed either more indicators of despair-related diseases or cardiometabolic risk, but not both….”
5. Sensitivity analyses (Supplemental Tables 4 and 5): In corrected sensitivity analyses testing associations of adolescent psychopathology with midlife diseases of despair among all Wave V respondents, we continue to observe statistically-significant associations for any adolescent psychopathology, internalizing psychopathology, and number of adolescent mental-health conditions. Corrected estimates of association for any psychopathology, internalizing psychopathology, and number of mental health conditions: adjusted IRRs=1.71 [1.57-1.85], 1.78 [1.63-1.93], and 1.48 [1.40-1.55], respectively. In corrected sensitivity analyses limiting to only prospective measures of adolescent psychopathology, we continue to observe statistically-significant associations with midlife diseases of despair. Corrected estimates of association for any psychopathology and CES-D-assessed depression: baseline IRRs=1.67 [1.44-1.90] and 1.66 [1.39-1.93], respectively; adjusted IRRs=1.61 [1.39-1.82] and 1.59 [1.32-1.86], respectively. Regarding associations with midlife cardiometabolic risk, the original association with any prospectively-assessed psychopathology was not statistically significant after adjustment; it is significant in corrected analyses (baseline IRR=1.13 [1.03-1.23], adjusted IRR=1.11 [1.01-1.20]). Consistent with previous findings, prospective CES-D-assessed depression is not significantly associated in corrected analyses (baseline IRR=1.13 [0.995-1.26], adjusted IRR=1.10 [0.97-1.23]).

No other analyses were impacted by this error, including (a) associations of adolescent physical health with midlife diseases of despair and cardiometabolic risk, (b) associations of externalizing psychopathology with midlife diseases of despair and cardiometabolic risk, and (c) the association of midlife diseases of despair with cardiometabolic risk.

We would also like to clarify the methods used to construct the indicator for adolescent conduct disorder. Supplemental Table 1 incorrectly stated that item responses were combined across waves. Questions from the delinquency scale were available at Waves I and II, but only Wave I included questions about skipping school without an excuse. As a result, our assessment of conduct disorder was limited to Wave I.

The authors apologize for these errors.
